# Supplementary material for: Association between Oncostatin M Expression and Inflammatory Phenotype in Experimental Arthritis Models and Osteoarthritis Patients
Source: Cells. 2021 Feb 27;10(3):508. doi: 10.3390/cells10030508 (PMC7997294; doi:10.3390/cells10030508)
Supplement: Supplementary file 1 [file cells-10-00508-s001.pdf]

Supplementary Information

Association between Oncostatin M expression and inflammatory phenotype in experimental arthritis models and osteoarthritis patients

Joao Pedro Garcia [1], Lizette Utomo [2,3], Imke Rudnik-Jansen [1], Jie Du [1], Nicolaas P. A. Zuithoff [4], Anita Krouwels [1], Gerjo J.V.M. van Osch [5,6] and Laura B. Creemers [1,*]

[1] Department of Orthopedics, University Medical Centre Utrecht, 3584 CX Utrecht, The Netherlands; j.p.marquesgarcia-2@umcutrecht.nl (J.P.G.); jansen.imke@gmail.com (I.R.-J.); j.du-2@umcutrecht.nl (J.D.); A.Krouwels-2@umcutrecht.nl (A.K.)
[2] Department of Oral and Maxillofacial Surgery & Special Dental Care, University Medical Center Utrecht, 3584 CX Utrecht, The Netherlands; l.utomo-2@umcutrecht.nl
[3] Department of Clinical Sciences, Faculty of Veterinary Medicine, Utrecht University, 3584 CL Utrecht, The Netherlands;
[4] Julius Center for Health Sciences and Primary Care, University Medical Center Utrecht, 3584 CX Utrecht, The Netherlands; NPAZuithoff@umcutrecht.nl
[5] Department of Orthopaedic Surgery, Erasmus MC, University Medical Center Rotterdam, 3015 GD Rotterdam, The Netherlands; g.vanosch@erasmusmc.nl
[6] Department of Otorhinolaryngology, Erasmus MC, University Medical Center Rotterdam, 3015 GD Rotterdam, The Netherlands
* Correspondence: l.b.creemers@umcutrecht.nl

Citation: Lastname, F.; Lastname, F.; Lastname, F. Title. *Cells* **2021**, *10*, x. https://doi.org/10.3390/xxxxx

Academic Editor: Alessandra Colombini

Received: 18 January 2021
Accepted: 23 February 2021
Published: date

Publisher's Note: MDPI stays neutral with regard to jurisdictional claims in published maps and institutional affiliations.

Copyright: © 2021 by the authors. Submitted for possible open access publication under the terms and conditions of the Creative Commons Attribution (CC BY) license (http://creativecommons.org/licenses/by/4.0/).

Table 1. Reliability assessment between PGPS samples stained with and without antigen retrieval.

Structure	Intra-class correlation	Confidence Interval (95 %)	
		Lower limit	Upper limit
**Cartilage**	0.870	0.671	0.945
Periosteum	0.919	0.826	0.962
Synovium	0.922	0.536	0.987
Meniscus	0.893	0.584	0.973
Ligaments	0.975	0.805	0.996

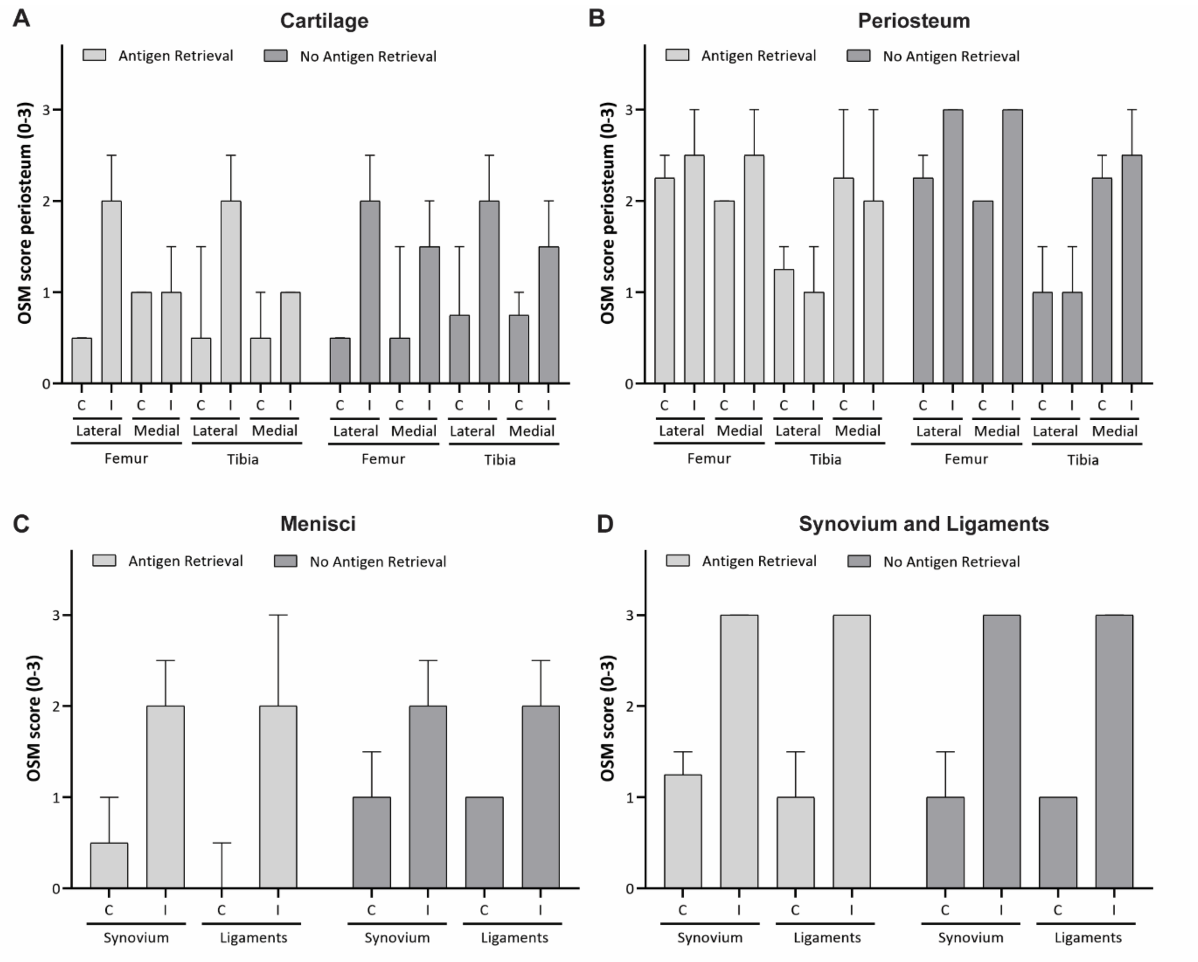

Figure 1. Effect of antigen retrieval on OSM scoring in the PGPS model samples**.** OSM score per anatomical structure: cartilage (**a**), periosteum (**b**), meniscus (**c**), synovium, and ligaments (**d**). Control (C), Induced (I). 0 = no staining, 1 = slight staining, 2 = moderate staining, 3 = extensive staining. Data are presented as median and 95 % CI.

Due to limited availability of samples, seven samples from the PGPS model were stained for OSM with and without antigen retrieval. OSM was scored as described before (0-3) for cartilage, periosteum, synovium, meniscus and ligaments. Scoring was done in a random order by two independent observers blinded for treatment and staining method, and scores were averaged.

The intra-class correlation coefficient (ICC) was used to calculate the agreement in OSM score between the two staining methods. A two-way mixed-effect model based on a mean-rating (k=2) and absolute agreement was used. ICC estimates and the 95% confidence intervals (CI) were reported. The ICC values are above 0.870 for all the joint structures, indicating that antigen retrieval does not affect the scoring for the PGPS model. Thus, the samples without antigen retrieval were used.

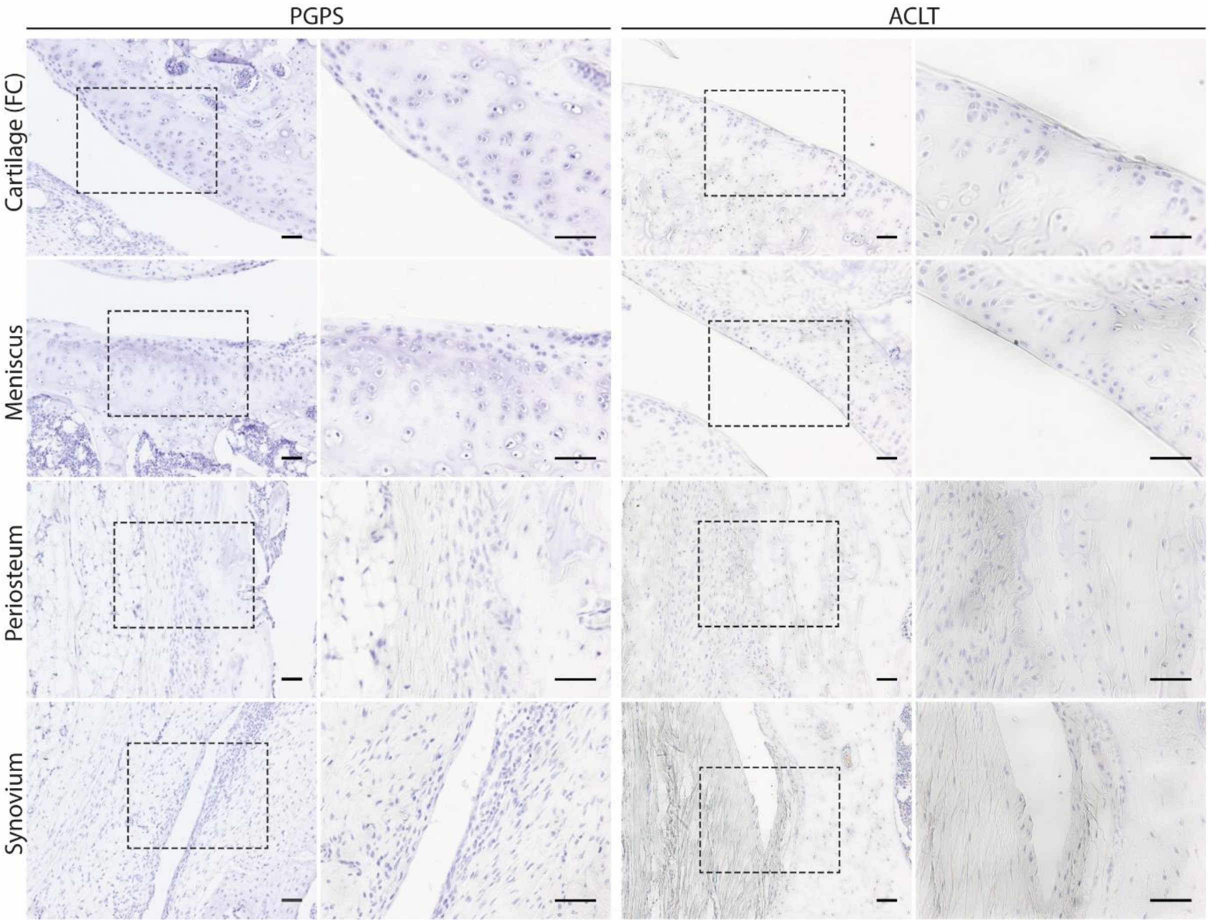

Figure 2. Negative control with mouse IgG isotype. Representative pictures of different joint structures: Femur condyle (FC) cartilage, meniscus, periosteum and synovium. Scale bar: 50 µm.

Table 2. Joint compartments.

Structure	Compartment
**Cartilage - chondrocytes**	Lateral femoral condyle
	Medial femoral condyle
	Lateral tibia plateau
	Medial tibia plateau
**Periosteum**	Lateral femur
	Medial femur
	Lateral tibia
	Medial tibia
**Meniscus**	Lateral
	Medial
**Synovium**	Femorotibial synovium
**Ligaments**	Collateral ligaments

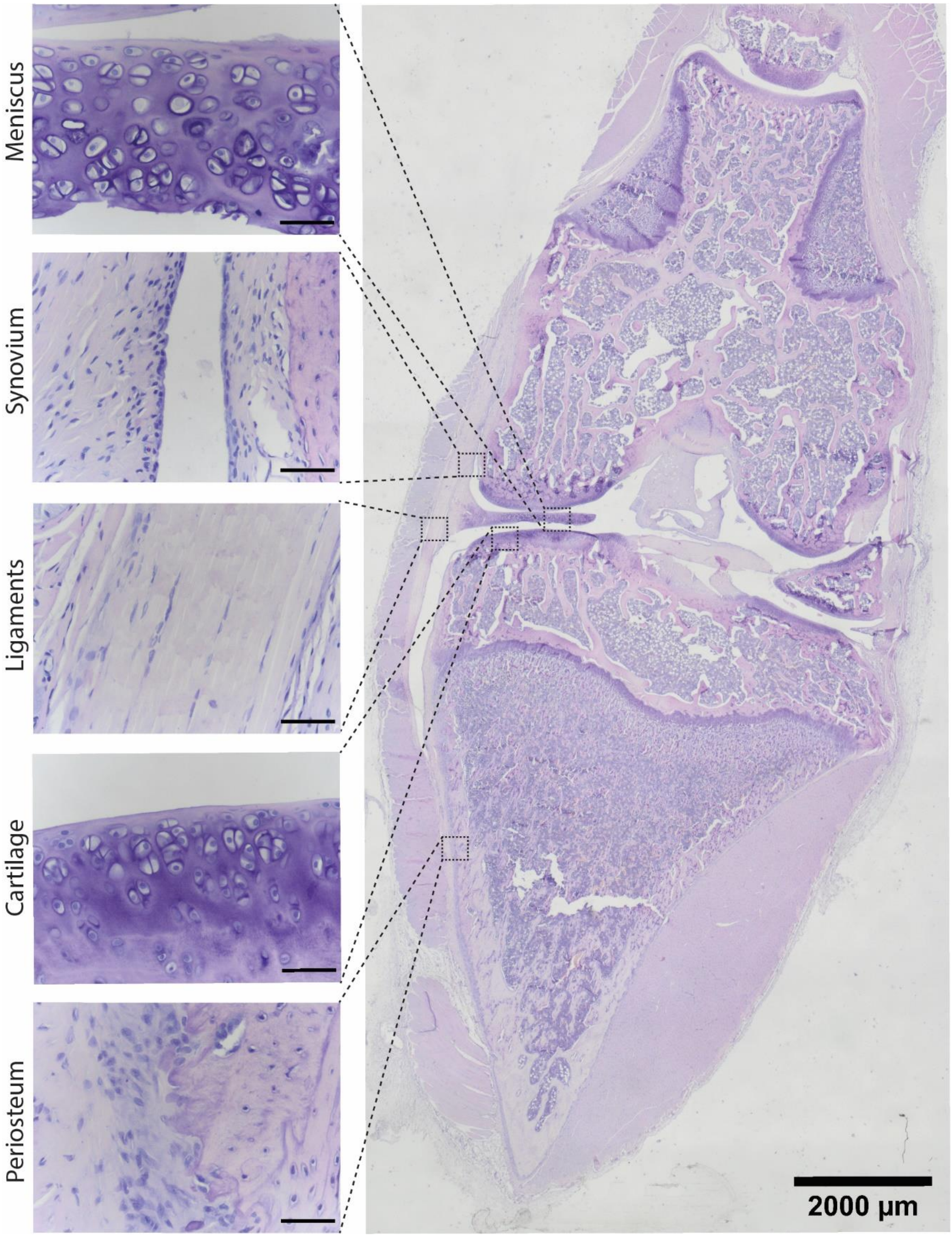

Figure 3. Hematoxylin & eosin staining of a rat joint**.** Histological overview of the rat joint with higher magnification pictures of the (medial side) compartments. For scoring purposes both medial and lateral compartments were taken into account. Scale bar = 50 µm (figures on the left).

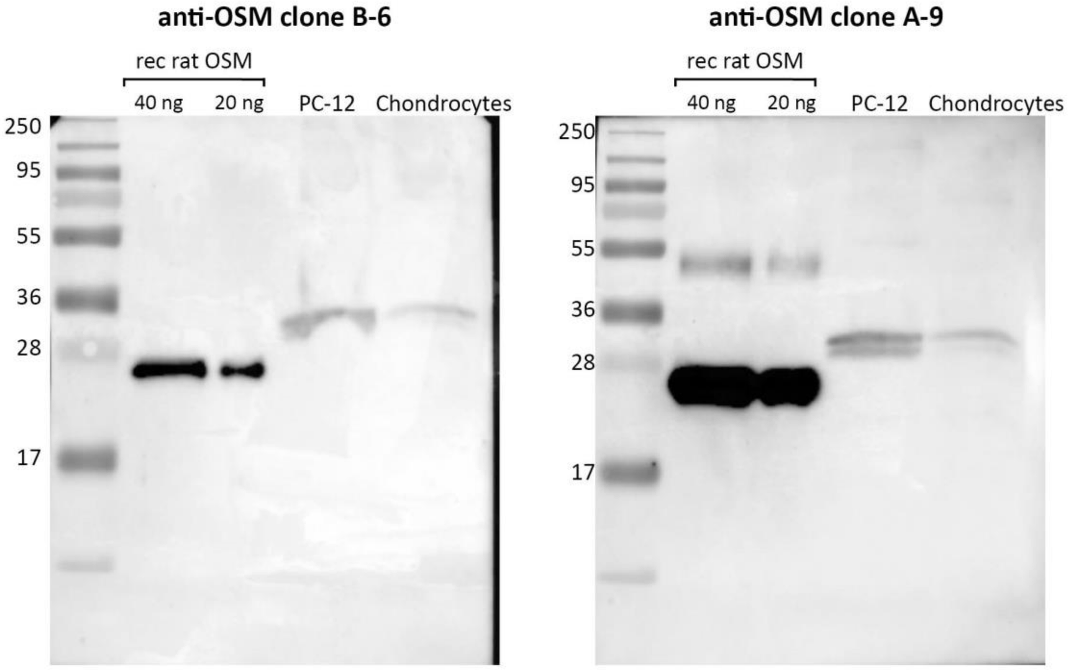

Molecular weights (kDa)	**Clone B-6**	**Clone A-9**
Recombinant rat OSM (40 ng)	26.17	25.38
Recombinant rat OSM (20 ng)	25.95	25.49
PC-12	33.95	32.10
Primary rat chondrocytes	34.52	32.87

Figure 4. Western blot analysis showing binding of two different anti-OSM antibodies to the OSM protein recombinant rat OSM, PC-12 cells, and primary rat chondrocytes.

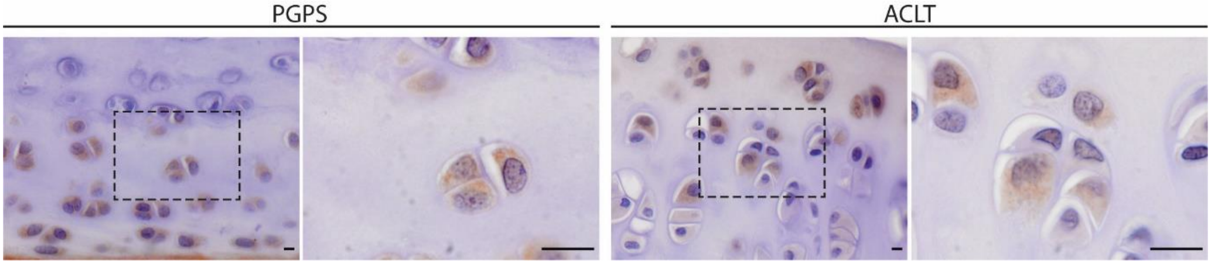

Figure 5. Intracellular OSM expression in chondrocytes in the PGPS and ACLT models. Scale bar: 10 μm.

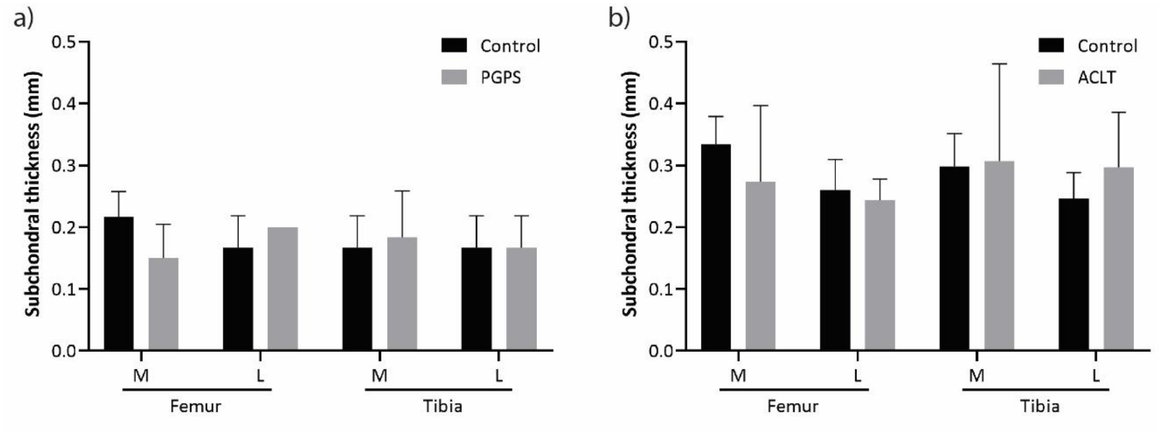

Figure 6. Subchondral bone sclerosis**.** Subchondral bone thickness measured by μCT in the PGPS (**a**) and ACLT + pMMx (**b**) models. Subchondral bone thickness was measured in the medial and lateral compartments of both femur condyle and tibia plateau.

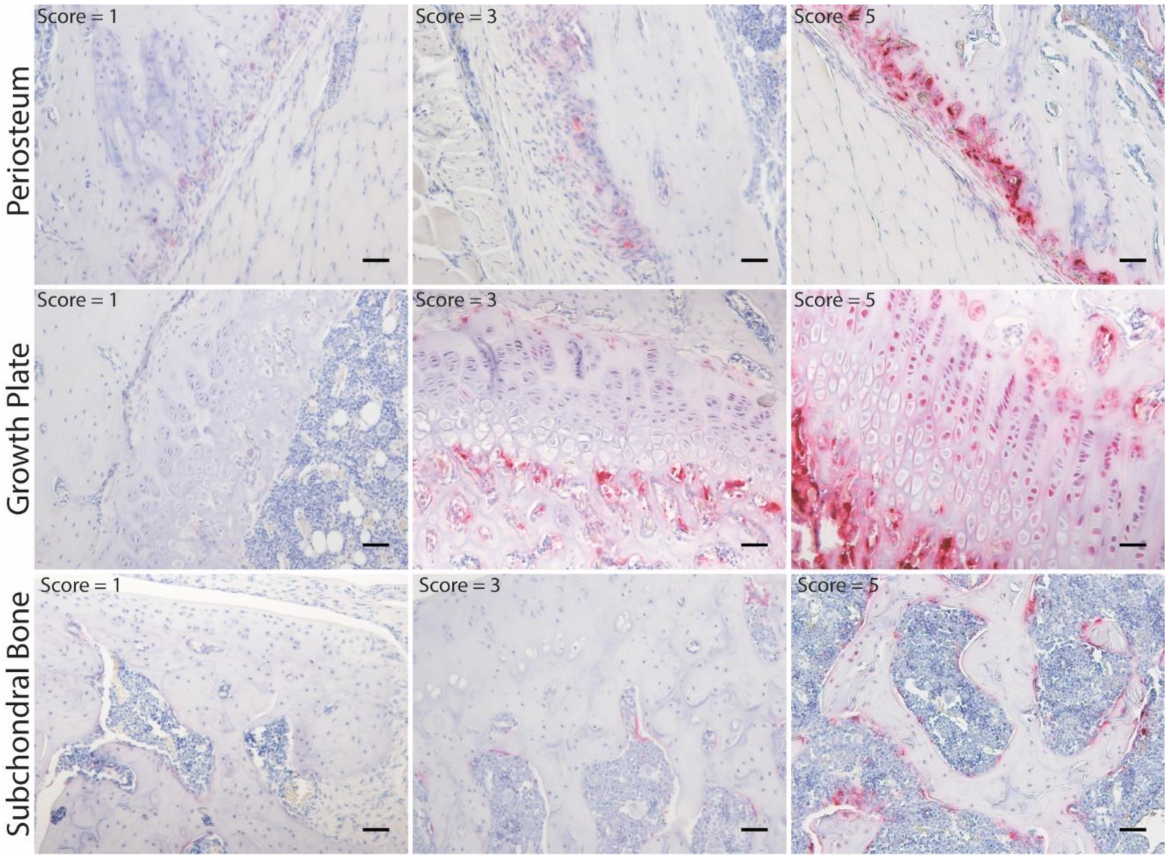

Figure 7. Trap staining**.** Representative pictures of different joint structures quantified for TRAP staining: periosteum, growth plate and subchondral bone.

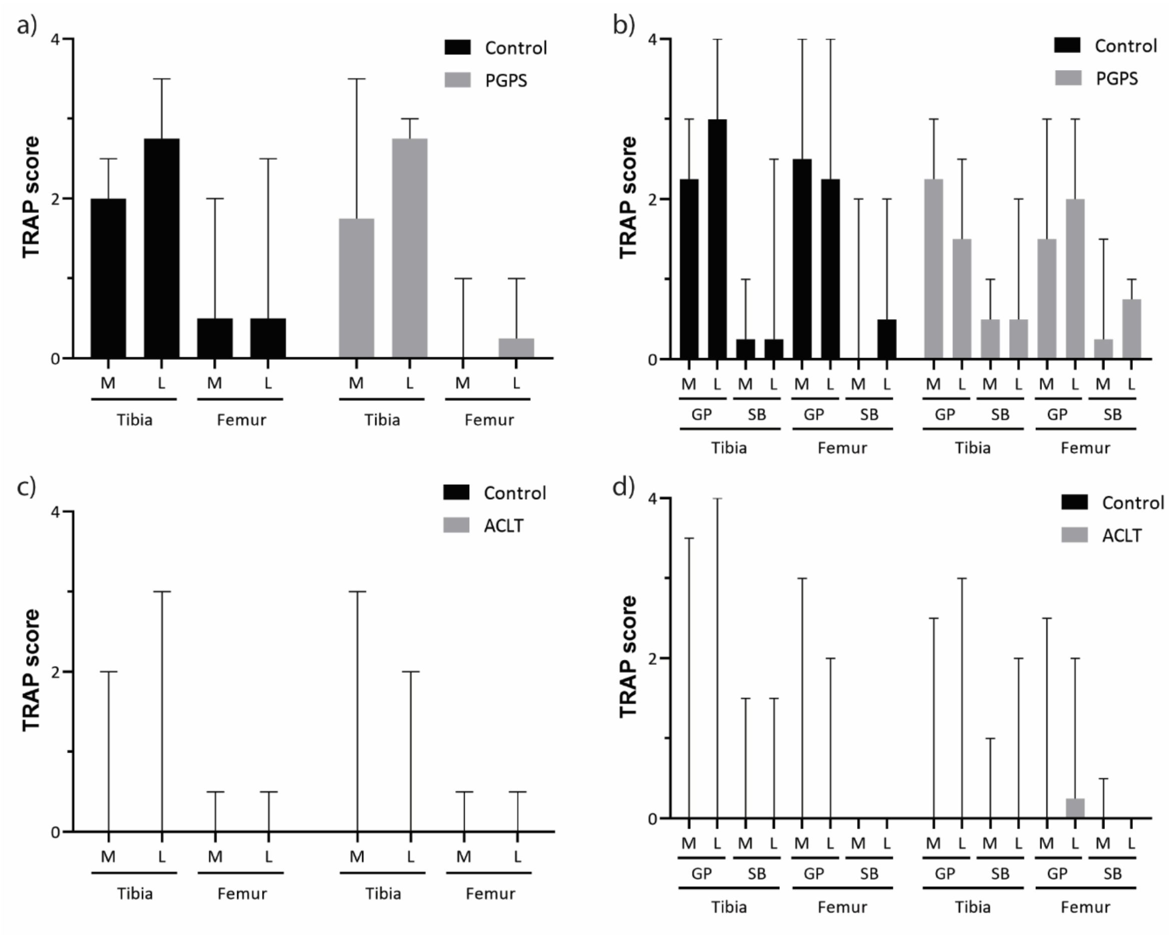

Figure 8. Quantification of TRAP staining**.** TRAP staining was performed in the knee sections of the PGPS (a and b), and ACLT + pMMx (c and d) models. TRAP staining was scored in periosteum (a and c), growth plate (GP) and subchondral bone (SB) (b and d) for tibia and femur in both medial (M) and lateral (L) compartments. Scoring was done in a random order by two independent observers blinded for treatment (JPG, AK), and scores were averaged. Data are presented as median and 95% CI.

Table 3. Spearman's correlation between OSM and remaining cytokines.

	**OSM**	
**Cytokines**	**Spearman's rho**	***p* value**
**IL-1α**	0.461	0.016*
**IL-1β**	0.193	0.335
**IL-4**	0.289	0.144
**IL-6**	0.350	0.074
**IL-8**	0.035	0.863
**IL-7**	0.450	0.019*
**IL-10**	0.106	0.599
**IL-13**	0.359	0.066
**TNF-α**	0.481	0.011*
**IFN-γ**	0.573	0.002*
**IL-1Ra**	0.038	0.852

Table 4. Differences in cytokine concentration between patient groups (OSM- and OSM+).

Cytokine	Difference	Sig.	95% Confidence Interval	
			Upper	Lower
**IFN-γ**	**−94.90**	**0.001**	**−39.43**	**−217.65**
**IL-1α**	**−20.91**	**0.003**	**−8.93**	**−45.78**
**TNF-α**	**−4.35**	**0.017**	**−1.28**	**−13.30**
**IL-7**	−4.00	0.050	−0.97	−14.03
**IL-4**	−1.64	0.052	−0.61	−3.66
**IL-6**	−1743.25	0.094	−150.13	−16669.00
**IL-13**	−18.74	0.097	−5.13	−52.89
**IL-1β**	−6.92	0.291	−0.62	−33.31
**IL-1Ra**	557.27	0.399	968.29	27.67
**IL-8**	47.28	0.399	27.90	7.05
**IL-10**	−4.05	0.595	0.59	−38.39
